# Association Between Sodium-Glucose Cotransporter-2 (SGLT2) Inhibitor Use and All-Cause Mortality in Patients With Pulmonary Arterial Hypertension

**DOI:** 10.7759/cureus.89465

**Published:** 2025-08-06

**Authors:** Irakli Lemonjava, Nino Gudushauri, Irakli Tskhakaia, Jose M Manzano, Apoorva Subramanian, Kevin B Lo, Zurab Azmaiparashvili

**Affiliations:** 1 Internal Medicine, Einstein Medical Center Philadelphia, Philadelphia, USA; 2 Pulmonary and Critical Care Medicine, Brigham and Women's Hospital, Harvard Medical School, Boston, USA; 3 Cardiology, Brigham and Women's Hospital, Harvard Medical School, Boston, USA

**Keywords:** idiopathic pulmonary arterial hypertension, innovative therapies, large database analysis, mortality benefit, sodium-glucose cotransporter-2 (sglt2) inhibitors

## Abstract

Introduction: Sodium-glucose cotransporter-2 inhibitors (SGLT2i) reduce mortality in heart failure patients with reduced and preserved ejection fraction. Their potential benefits in pulmonary arterial hypertension (PAH) are unknown. This study evaluates the relationship between SGLT2i use and all-cause mortality in patients with PAH.

Materials and methods: Using the TriNetX Research Network, we included patients diagnosed with PAH after January 1, 2013. Group A (SGLT2i group) included patients prescribed canagliflozin, dapagliflozin, empagliflozin, or ertugliflozin, and Group B (non-SGLT2i group) included patients not using these medications. Outcomes were assessed at one-, three-, and five-year intervals following the index event (PAH diagnosis and medication initiation). Propensity score matching was used to account for demographics and comorbidities. Matched cohorts included 7,998 patients in Group A and 8,006 in Group B.

Results: Over one year, mortality occurred in 614 of 7,998 patients (7.7%) in Group A, whereas Group B experienced 1,251 deaths among 8,006 patients (15.6%), yielding a risk difference of 7.9% (p < 0.0001). This mortality advantage associated with SGLT2i inhibitor use remained evident at the three-year mark, with 1,012 of 7,998 patients (12.7%) deceased in Group A compared to 1,930 of 8,006 (24.1%) in Group B, translating to an 11.5% absolute risk difference (p < 0.0001). By five years, mortality had reached 1,139 out of 7,998 patients (14.2%) in Group A and 2,190 out of 8,006 (27.4%) in Group B, with a corresponding risk difference of 13.1% (p < 0.0001).

Conclusions: SGLT2i use was associated with significant and sustained mortality reduction in PAH patients. A 13% absolute risk reduction at five years highlights the potential for a transformative impact on PAH management. Randomized controlled trials are warranted to confirm these findings and guide clinical practice.

## Introduction

Pulmonary arterial hypertension (PAH) is a progressive and life-threatening condition characterized by a complex interplay of metabolic, inflammatory, and hemodynamic alterations that drive pulmonary vascular remodeling. These changes lead to elevated pulmonary vascular resistance and pulmonary arterial pressures, ultimately resulting in right ventricular failure, increased morbidity, and early mortality [1]. Despite significant advances in understanding PAH pathophysiology, the disease remains devastating, with a high symptom burden and limited long-term treatment options [2]. Currently available therapeutic agents, primarily pulmonary vasodilators, may improve functional status and exercise capacity but have shown limited ability to reduce mortality [3]. As such, there is growing interest in repurposing agents with pleiotropic effects that target the metabolic and inflammatory pathways implicated in PAH progression. Several studies have noted an association between PAH and insulin resistance, type 2 diabetes mellitus, and metabolic syndrome, prompting exploratory trials using antidiabetic agents such as metformin, which have shown early signals of benefit [4]. One class of medications that has garnered substantial interest is the sodium-glucose cotransporter-2 inhibitors (SGLT2i). These agents, initially developed for glycemic control, have since demonstrated robust reductions in morbidity and mortality in patients with heart failure and chronic kidney disease, regardless of diabetes status [5,6]. Given their favorable safety profile, cardiometabolic effects, and increasing global accessibility, SGLT2i represents a promising therapeutic adjunct in PAH [7]. However, its impact on long-term outcomes in this population remains unclear. In this study, we investigate the association between SGLT2i use and all-cause mortality in patients with PAH using a large, real-world database.

## Materials and methods

Study design and data source

This retrospective cohort study was conducted using data from the TriNetX Research Network. This global federated health research platform aggregates de-identified electronic medical records from over 95 healthcare organizations, encompassing more than 130 million patients. As the data are de-identified and Health Insurance Portability and Accountability Act-compliant, this study was exempt from institutional review board approval. TriNetX provides access to diagnoses (ICD-10-CM), procedures (CPT), medications (RxNorm), and laboratory results through an integrated analytic interface.

Study population

We included adult patients aged 18 years or older with a diagnosis of PAH, identified using ICD-10-CM codes consistent with Group 1 PAH, as outlined in the Appendices section. The study period spanned from January 1, 2013, to October 10, 2024, during which relevant clinical and prescription data were extracted.

Patients were stratified into two cohorts based on exposure to SGLT2i. The SGLT2i cohort (Group A) comprised patients with a recorded diagnosis of PAH who had received at least one prescription for an SGLT2i, identified through corresponding RxNorm codes. For these individuals, the index date was defined as the date of the first recorded SGLT2i prescription. The control cohort (Group B) included patients with a diagnosis of PAH and no documented exposure to SGLT2i during the study period. To minimize immortal time bias, index dates in the control group were randomly assigned to mirror the temporal distribution of index dates observed in the SGLT2i group.

Patients were excluded if they had received an SGLT2i before the assigned index date or if key demographic information was missing from the dataset.

Study outcomes

The primary outcome was all-cause mortality, determined using the "deceased" status flag in TriNetX. Patients already marked as deceased before the index date were excluded. As TriNetX mortality data may be incomplete for outpatient deaths, this outcome reflects deaths documented within the health systems contributing data.

Covariates and propensity score matching

Covariates included age, sex, race/ethnicity, comorbidities (grouped by ICD-10 chapters: circulatory, endocrine, respiratory, musculoskeletal, nervous, digestive, hematologic, and genitourinary systems), social determinants (Z codes), and relevant laboratory values (e.g., hemoglobin A1C, BNP). Medication use, including pulmonary vasodilators, was also captured using RxNorm codes. We applied 1:1 propensity score matching using a nearest-neighbor algorithm with a caliper of 0.1 to balance baseline characteristics between groups. Covariate balance was assessed using standardized mean differences, with values <0.1 indicating adequate balance.

Statistical analysis

Descriptive statistics were used to summarize baseline characteristics. Continuous variables were expressed as mean ± standard deviation (SD) and compared using independent t-tests. Categorical variables were presented as counts and percentages and compared using chi-square or Fisher’s exact tests, as appropriate. After matching, we compared the incidence of mortality between the two groups and calculated absolute risk reduction, relative risk reduction, risk ratio, and 95% confidence intervals. All hypothesis testing was two-tailed, with a p-value < 0.05 considered statistically significant.

## Results

A total of 8,400 patients with PAH who received an SGLT2i and 133,056 patients without SGLT2i exposure were included in the initial cohort. Before propensity score matching, patients in the SGLT2i group were younger (mean age 68.0 vs. 71.8 years, p < 0.01) and had a greater burden of cardiovascular, respiratory, and endocrine comorbidities (all p < 0.01). They were more frequently treated with pulmonary vasodilators (all p < 0.01, except Bosentan, p = 0.06), had higher mean hemoglobin A1C (6.97% vs. 6.37%, p < 0.01), and lower concentrations of BNP (609 vs. 723 pg/mL, p < 0.05) and NT-proBNP (2,618 vs. 4,225 pg/mL, p < 0.01).

After 1:1 propensity score matching, 8,349 patients remained in each group. The matched cohorts were well balanced regarding sex, race, comorbidity profiles, and pulmonary vasodilator use. Small residual differences remained, with the SGLT2i group slightly older (68.4 vs. 67.1 years, p < 0.01), demonstrating higher mean hemoglobin A1C (7.01% vs. 6.14%, p < 0.01) and lower concentrations of BNP (617 vs. 877 pg/mL, p < 0.05) and NT-proBNP (2,610 vs. 4,242 pg/mL, p < 0.01). The most frequently prescribed pulmonary vasodilators in both groups included sildenafil, tadalafil, ambrisentan, and treprostinil (Table 1).

**Table 1:** Baseline characteristics and propensity score matching SGLT2i: sodium-glucose cotransporter-2 inhibitor, SD: standard deviation, ICD: International Classification of Diseases

Characteristics	SGLT2i group (N = 8,384)	Non-SGLT2i group (N = 132,944)	Standardized mean difference		p-value	SGLT2i group (N = 8,349)	Non-SGLT2i group (N = 8,349)	Standardized mean difference	p-value
Before propensity score matching						After propensity score matching			
Age, mean ± SD	68 ± 13.8	71.8 ± 16.6		0.25	<0.01	68.4 ± 13.6	67.1 ± 17.9	0.08	<0.01
Female subjects, %	53.9	60		0.12	<0.01	54.7	54.6	0.02	0.88
Hispanic, %	23.8	30.1		0.03	<0.01	24	23.6	0.008	0.61
White, %	58.4	59.7		0.02	<0.05	58.8	59.3	0.01	0.5
Black, %	23.2	17.8		0.13	<0.01	22.8	22.3	0.01	0.5
Asian, %	3.9	3.2		0.03	<0.01	3.9	4	0.005	0.8
Unknown, %	14.5	19.3		0.11	<0.01	14.5	14.4	0.007	0.4
Diagnoses (%)									
Cardiovascular disorders	99	80.4		0.64	<0.01	99	98.9	0.009	0.6
Endocrine disorders	96.2	64.9		0.85	<0.01	96	95.6	0.01	0.2
Factors influencing health status (ICD-10 codes Z00-Z99)	92.2	67.5		0.64	<0.01	92	91.8	0.007	0.6
Respiratory disorders	87.9	59.7		0.7	<0.01	87.5	87.2	0.009	0.6
Musculoskeletal disorders	85.4	58.5		0.67	<0.01	85.4	83	0.04	0.01
Nervous system disorders	83.7	48.9		0.79	<0.01	83.1	82.9	0.005	0.72
Gastrointestinal disorders	82.9	51.9		0.71	<0.01	82.5	82.1	0.008	0.6
Genitourinary disorders	81.3	49.1		0.71	<0.01	80.7	79.4	0.03	0.05
Hematologic disorders	71.9	40.2		0.7	<0.01	71.3	70.3	0.02	0.19
Diagnoses not elsewhere classified (ICD-10 codes R 00-R 99)	97.4	78.1		0.61	<0.01	97.3	97	0.01	0.3
Medications (%)									
Sildenafil	19.9	7.4		0.37	<0.01	18.6	18.4	0.003	0.8
Tadalafil	10.07	3.2		0.27	<0.01	9	9.1	0.001	0.9
Ambrisentan	4	1.7		0.13	<0.01	3.6	3.8	0.002	0.9
Treprostinil	3.5	1.04		0.16	<0.01	3.2	3.3	0.005	0.8
Epoprostenol	2.3	0.8		0.12	<0.01	2.2	1.9	0.02	0.3
Riociguat	1.8	0.6		0.1	<0.01	1.8	1.9	0.01	0.5
Bosentan	1	0.8		0.02	0.06	0.9	0.6	0.02	0.12
Iloprost	0.3	0.1		0.04	<0.01	0.3	0.2	0.02	0.3
Laboratory parameters (mean ± SD)									
Hemoglobin A1C (%)	6.97 ± 1.78	6.37 ± 1.53		0.36	<0.01	7.01 ± 1.79	6.14 ± 1.43	0.5	<0.01
Brain natriuretic peptide in pg/mL	609 ± 2071	723 ± 2837		0.04	<0.05	617 ± 2106	877 ± 3975	0.08	<0.01
N-terminal pro-brain natriuretic peptide in pg/mL	2618 ± 5244	4225 ± 9063		0.21	<0.01	2610 ± 5089	4242 ± 9394	0.2	<0.01

In the matched cohort, the SGLT2i group demonstrated a significantly lower all-cause mortality rate compared to controls. Over one year, mortality occurred in 614 of 7,998 patients (7.7%) in Group A, whereas Group B experienced 1,251 deaths among 8,006 patients (15.6%), yielding a risk difference of 7.9% (p < 0.0001). This mortality advantage associated with SGLT2i use remained evident at the three-year mark, with 1,012 of 7,998 patients (12.7%) deceased in Group A compared to 1,930 of 8,006 (24.1%) in Group B, translating to an 11.5% absolute risk difference (p < 0.0001). By 5 years, mortality had reached 1,139 out of 7,998 patients (14.2%) in Group A and 2,190 out of 8,006 (27.4%) in Group B, with a corresponding risk difference of 13.1% (p < 0.0001) (Table 2).

**Table 2:** Mortality risk in PAH with and without SGLT2i use PAH: pulmonary arterial hypertension, SGLT2i: sodium-glucose cotransporter-2 inhibitor

Outcome	Groups	Overall risk, %	Absolute risk reduction, %	Overall risk, %	Absolute risk reduction, %
Before propensity score matching				After propensity score matching	
All-cause mortality at 1 year	PAH on SGLT2i	7.6 (632/8,270)	4.53 (3.9-5), p < 0.01	7.7 (614/7,998)	7.9 (6.7-8.9), p < 0.01
	Not on SGLT2i	12.2 (14,364/117,993)		15.6 (1,251/8,006)	
All-cause mortality at 3 years	PAH on SGLT2i	12.5 (1,035/8,270)	10.04 (9.2-10.8), p < 0.01	12.7 (1,012/7,998)	11.5 (10.3-12.6), p < 0.01
	Not on SGLT2i	22.6 (26,614/117,993)		24.1 (1,930/8,006)	
All-cause mortality at 5 years	PAH on SGLT2i	14 (1,152/8270)	15.3 (14.5-16), p < 0.01	14.2 (1,139/7,998)	13.1 (11.9-14.4), p < 0.01
	Not on SGLT2i	29.3 (34,482/117,782)		27.4 (2,190/8,006)	

## Discussion

In this large retrospective cohort analysis, we found that patients with PAH who were exposed to SGLT2i experienced significantly reduced all-cause mortality at one-, three-, and five-year follow-up intervals. These findings are notable, as they suggest that the benefits of SGLT2i may extend beyond glycemic control into the realm of pulmonary vascular disease. Although initially introduced for the treatment of type 2 diabetes mellitus, the clinical utility of SGLT2i has broadened considerably over the past decade. They are now considered a foundational therapy in patients with heart failure, regardless of ejection fraction status, reflecting their systemic benefits beyond glucose lowering [8]. Their potential in PAH may follow a similar trajectory, particularly given the overlap in cardiometabolic pathophysiology [7].

While SGLT2i are primarily recognized for their glucose-lowering effects and osmotic diuresis, preclinical studies have increasingly demonstrated their broader biological activity through modulation of key intracellular signaling pathways, notably the Notch and mitogen-activated protein (MAP) kinase cascades. These pathways are essential regulators of vascular remodeling, cellular proliferation, apoptosis, and maintenance of homeostasis within the pulmonary vasculature [9,10].

The Notch signaling pathway governs cellular differentiation, proliferation, and survival, and plays a vital role in the endothelium and smooth muscle of the pulmonary arterial wall. In PAH, aberrant activation of this pathway, particularly via Notch3, promotes smooth muscle cell hyperproliferation and vascular remodeling [11]. Preclinical data suggest that SGLT2i may attenuate these pathological changes by suppressing excessive Notch3 signaling, thereby mitigating vascular remodeling and its associated hemodynamic consequences [10].

Similarly, the MAP signaling cascade, which includes key kinases involved in the cellular response to stress, inflammatory stimuli, and growth factors, is frequently upregulated in pulmonary hypertension. Its activation promotes inflammatory cell recruitment, smooth muscle proliferation, and vascular hypertrophy [12]. SGLT2i have been shown to downregulate components of the MAP kinase pathway, leading to reduced inflammation and cellular proliferation within the pulmonary circulation [9].

In addition to the previously described mechanisms, emerging preclinical evidence indicates that SGLT2i may confer vascular protective effects through distinct pathways, including the enhancement of nitric oxide signaling and attenuation of oxidative stress. Experimental models have demonstrated that SGLT2i increases nitric oxide bioavailability, reduces reactive oxygen species, and exerts anti-inflammatory effects. These actions have been associated with the prevention of maladaptive pulmonary vascular remodeling in experimental models of pulmonary hypertension [13]. However, the translational significance of these findings remains uncertain, as off-target pharmacologic effects may contribute to the observed outcomes. Furthermore, in vitro and in vivo studies have shown that SGLT2i support endothelial integrity by mitigating oxidative injury, promoting nitric oxide-mediated vasodilation, and suppressing pro-inflammatory signaling cascades [14]. While these mechanisms are pathophysiologically relevant to PAH, the majority of supporting data are preclinical, and the impact of SGLT2i on angiogenesis and clinical endpoints in this population has not yet been established [14].

Together, these mechanistic insights support the hypothesis that became the foundation of our study, that SGLT2i may exert direct vasculoprotective effects in PAH, beyond their systemic hemodynamic benefits. By modulating molecular pathways implicated in disease progression, SGLT2i may represent a promising therapeutic adjunct in the management of pulmonary vascular disease.

This study has several significant limitations that should be considered when interpreting the findings. First, as a retrospective analysis of real-world electronic health record data, the study is inherently subject to residual confounding, despite our use of rigorous 1:1 propensity score matching. We did not have access to key clinical variables such as medication dosage, duration of therapy, or patient adherence, nor were lifestyle factors, such as smoking status, diet, or physical activity, available in the dataset. The diagnosis of PAH was based on ICD-10-CM coding consistent with Group 1 PAH, and we were unable to confirm these diagnoses through right heart catheterization or echocardiography. Furthermore, because definitions and coding practices may have evolved over the study period, some degree of diagnostic misclassification is possible.

In terms of treatment exposure, our analysis treated SGLT2i use as a class effect. Although all four agents (canagliflozin, dapagliflozin, empagliflozin, and ertugliflozin) were included, we did not stratify patients by individual drug, nor were we able to determine whether patients switched between agents over time. Therefore, we could not assess drug-specific effects or differences in safety profiles. Similarly, while we did include pulmonary vasodilator use as a covariate in the matching process, we could not distinguish between patients receiving monotherapy versus combination therapy or determine the severity of PAH based on clinical or hemodynamic staging.

Our study population was drawn from multiple healthcare systems across the world, and we applied matching by age, sex, race, and comorbid conditions to improve comparability. However, the generalizability of our findings to more diverse, global, or resource-limited settings remains uncertain. In addition, all-cause mortality was determined using the "deceased" status flag within TriNetX, which may underrepresent deaths that occurred outside of contributing health systems and lead to potential underestimation of true mortality rates.

Finally, this study is observational and retrospective. While the consistency and magnitude of the mortality reduction observed across multiple time points are compelling, our findings should be viewed as hypothesis-generating rather than definitive. Prospective, randomized controlled trials are needed to confirm the potential therapeutic benefit of SGLT2i in patients with PAH and to explore the underlying mechanisms that may account for these outcomes.

## Conclusions

These findings highlight a significant and durable survival benefit associated with SGLT2i use in patients with PAH. In this matched cohort analysis, the SGLT2i group consistently demonstrated lower all-cause mortality compared to controls across all time points evaluated. The mortality benefit was evident as early as one year and continued to widen through three and five years of follow-up, with absolute risk reductions of 7.9%, 11.5%, and 13.1%, respectively. These results contribute to the growing body of literature exploring the potential applications of SGLT2i beyond their established use in diabetes, chronic kidney disease, and heart failure. While the observed association between SGLT2i use and reduced all-cause mortality in patients with PAH is notable, these findings should be interpreted with caution. Given the retrospective design and reliance on administrative data, the results are best viewed as hypothesis-generating and not sufficient to infer causality. Further prospective studies, ideally randomized controlled trials, are essential to confirm these associations, explore potential underlying mechanisms, and better define which patient populations, if any, may benefit from SGLT2i therapy in the setting of pulmonary vascular disease.

## Appendices

Inclusion/exclusion criteria, matching criteria, and outcomes with corresponding ICD-10, RxNorm codes

Inclusion Criteria

ICD-10 codes: any of the following

I27.0 - pulmonary arterial hypertension (primary pulmonary hypertension)

AND 

RxNorm codes for

Canagliflozin (1373458) OR Dapagliflozin (1488564) OR Empagliflozin (1545653) OR Ertugliflozin (1992672)

Matching Criteria

ICD-10 Codes

I10-I99 - Diseases of the circulatory system

R00-R99 - Symptoms, signs and abnormal clinical and laboratory findings, not elsewhere classified

E00-E89 - Endocrine, nutritional and metabolic diseases

Z00-Z99 - Factors influencing health status and contact with health services

J00-J99 - Diseases of the respiratory system

M00-M99 - Diseases of the musculoskeletal system and connective tissue

G00-G99 - Diseases of the nervous system

K00-K95 - Diseases of the digestive system

N00-N99 - Diseases of the genitourinary system

D50-D89 - Diseases of the blood and blood-forming organs and certain disorders involving the immune mechanisms

RxNorm Codes

RxNorm -136411 - Sildenafil

RxNorm - 358263 - Tadalafil

RxNorm - 358274 - Ambrisentan

RxNorm - 343048 - Treprostinil

RxNorm - 8814 - Epoprostenol

RxNorm - 1439816 - Riociguat

RxNorm - 75207 - Bosentan

RxNorm - 40138 - Iloprost

TNX Curated Codes

TNX Curated - 9037- Hemoglobin A1C (in blood)

TNX Curated - 9003 - Natriuretic peptide B. (in serum, plasma, or blood)

TNX Curated - 9072 - Natriuretic peptide B. Prohormone N-terminal (in serum, plasma, or blood)
